# Functional outcome of scapulectomy in children – single-center experience and systematic review of the literature

**DOI:** 10.1016/j.xrrt.2025.01.004

**Published:** 2025-02-12

**Authors:** Tobias Jhala, Maximilian Holweg, Markus Dietzel, Jürgen Schäfer, Martin Ebinger, Justus Lieber, Jörg Fuchs

**Affiliations:** a Department of Pediatric Surgery and Pediatric Urology, University Children's Hospital Tuebingen, Tuebingen, Germany; b Department of Radiology, Diagnostic and Interventional Radiology, University Hospital Tuebingen, Tuebingen, Germany; c Department for General Pediatrics, Hematology and Oncology, University Children's Hospital Tuebingen, Children's Hospital, Tuebingen, Germany

**Keywords:** Pediatric, Tumor, Scapulectomy, Orthopedic surgery, Reconstruction, Functional outcome

## Abstract

**Background:**

Recent advances in reconstruction of the shoulder girdle and scapula had a significant impact on functional outcome in adults who underwent oncologic scapulectomy. In children and adolescents, scapula tumors are rare. Moreover, the growing skeleton and high functional demands in this age group may hinder transferability of the promising results achieved in adults. This study aims to explore the functional outcome and different reconstructive options used in children undergoing (partial) scapulectomy.

**Methods:**

A single-center retrospective analysis of scapula tumors in children was performed. Furthermore, a systematic review and synthesis of qualitative and quantitative studies were conducted to investigate the functional outcome of children and adolescents undergoing Malawer II or Malawer III resection.

**Results:**

In total, 3 patients were deemed eligible for the single-center retrospective analysis. The 3 children (2 boys, 1 girl, aged 4 – 11 years) all had Ewing sarcoma of the scapula. Two patients underwent Malawer II resection and had a better functional outcome than the 1 child that underwent Malawer III resection. Concerning the systematic review, of the 714 initial search results, 17 studies were eligible for inclusion. In total, 47 patients were extracted from the 17 studies. The analysis showed that patients who underwent Malawer III resection had a significantly better functional outcome if a reconstructive surgery was performed. Patients who underwent glenoid-preserving Malawer II resection showed similar results with or without reconstruction.

**Conclusion:**

Children and adolescents undergoing Malawer III resection benefit from a reconstructive procedure other than humeral suspension. Reconstruction using either endoprosthesis or extracorporeal irradiation and reimplantation provide similar functional outcome after Malawer III resection. In Malawer II resection, reconstructive procedures do not influence functional outcome.

Tumors of the scapula are rare, even more so in children. Only 1.6%-2.8% of bone tumors are reported to be localized in the scapular region, usually presenting between the second to sixth decades of life.^28^ Scapular tumors can grow quite large before being diagnosed, which can lead to tumor extension into the surrounding tissues; henceforth, function preserving surgery could be quite difficult. Partial or total scapulectomy are often required in advanced stage of the disease.^10^^,^^18^^,^^25^^,^^34^^,^^35^ The wide resection needed to optimize oncological outcome can lead to profound and long-lasting effects on function of the upper extremity.^7^^,^^34^

Historically, before the implementation of the Tikhoff–Linberg procedure in the 1970s, patients with scapular tumors often underwent forequater amputation. The Tikhoff–Linberg procedure preserved the upper extremity while achieving the same rates of tumor control and survival. However, it resulted in a flail joint that greatly affected shoulder and upper extremity function and cosmesis.^25^^,^^28^^,^^31^^,^^34^ Early on, the Tikhoff–Linberg procedure with humeral suspension (HS) was used, which evolved into complete endoprosthesis reconstruction of the proximal humerus, shoulder joint, and scapula.^27^ Today, at least in adults, limb sparing surgery and reconstructions other than HS have become the standard and have shown to provide good functional and oncological outcome.

Depending on the location of the primary tumor, a specific shoulder girdle resection (classified by Malawer) is indicated. Although Malawer II resection preserves the glenoid, in Malawer III resection, a total scapulectomy is performed which results in considerable deterioration of the shoulder function.^1^^,^^16^^,^^28^ None of the reconstructive procedures used (endoprosthesis, allograft, and extracorporeal irradiation and reimplantation [ECI+RP]) has been proven to be superior.^1^^,^^4^^,^^18^^,^^31^^,^^35^ Children and adolescents have high functional requests concerning the upper extremity, but reconstructive procedures are complicated by the dynamics of a growing skeleton.^4^^,^^11^ Regarding scapular tumors in children, only few case series and case reports exist in the literature. Moreover, only a little proportion of them describes the type of resection, reconstructive procedures, or functional outcome.^10^^,^^11^^,^^18^^,^^25^^,^^34^^,^^35^

Herein, we present a single-center experience with scapula tumors in children as well as a systematic review of the literature concerning this topic.

## Case presentation

**Figure 1:**
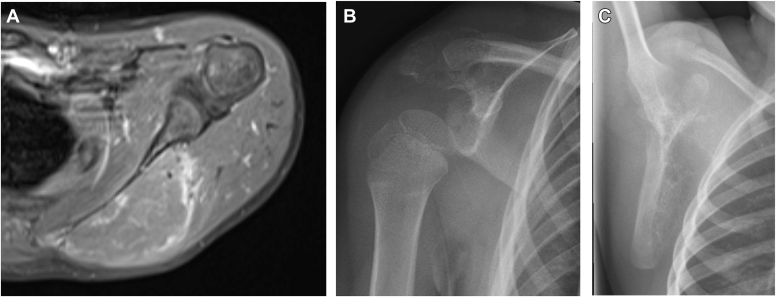
Initial imaging of the scapula tumor of patient 1-3. (**A**) Saggital magnetic resonance imaging (MRI) scan showing the Ewing sarcoma of the scapula body of patient 1. (**B**) Radiograph demonstrating the acromial Ewing sarcoma of patient 2. (**C**) Radiograph showing the Ewing sarcoma of the scapula body of patient 3.

A single-center retrospective data analysis of children undergoing partial or total scapulectomy was conducted. The analysis was approved by the local ethics committee. Three children (2 boys and 1 girl aged 4-11 years) were included; all had Ewing sarcoma (ES) of the scapula (initial imaging of patients 2 and 3 see Fig. 1).

Epidemiological data and oncologic and surgical therapy are displayed in Table I.

**Table I:** Single-center patient cohort.

Patients	Sex	Diagnosis	Age	Tumor location	Resection type	Follow-up [mo]	MSTS
1	m	ES	11	Body of scapula	Malawer II	6	28
2	m	ES	4	Acromion	Acromion resection	13	24
3	w	ES	4	Body of scapula	Malawer III & HS	238	16

*MSTS*, Musculoskeletal Tumor Society; *ES*, Ewing sarcoma; *HS*, humeral suspension.

**Figure 2:**
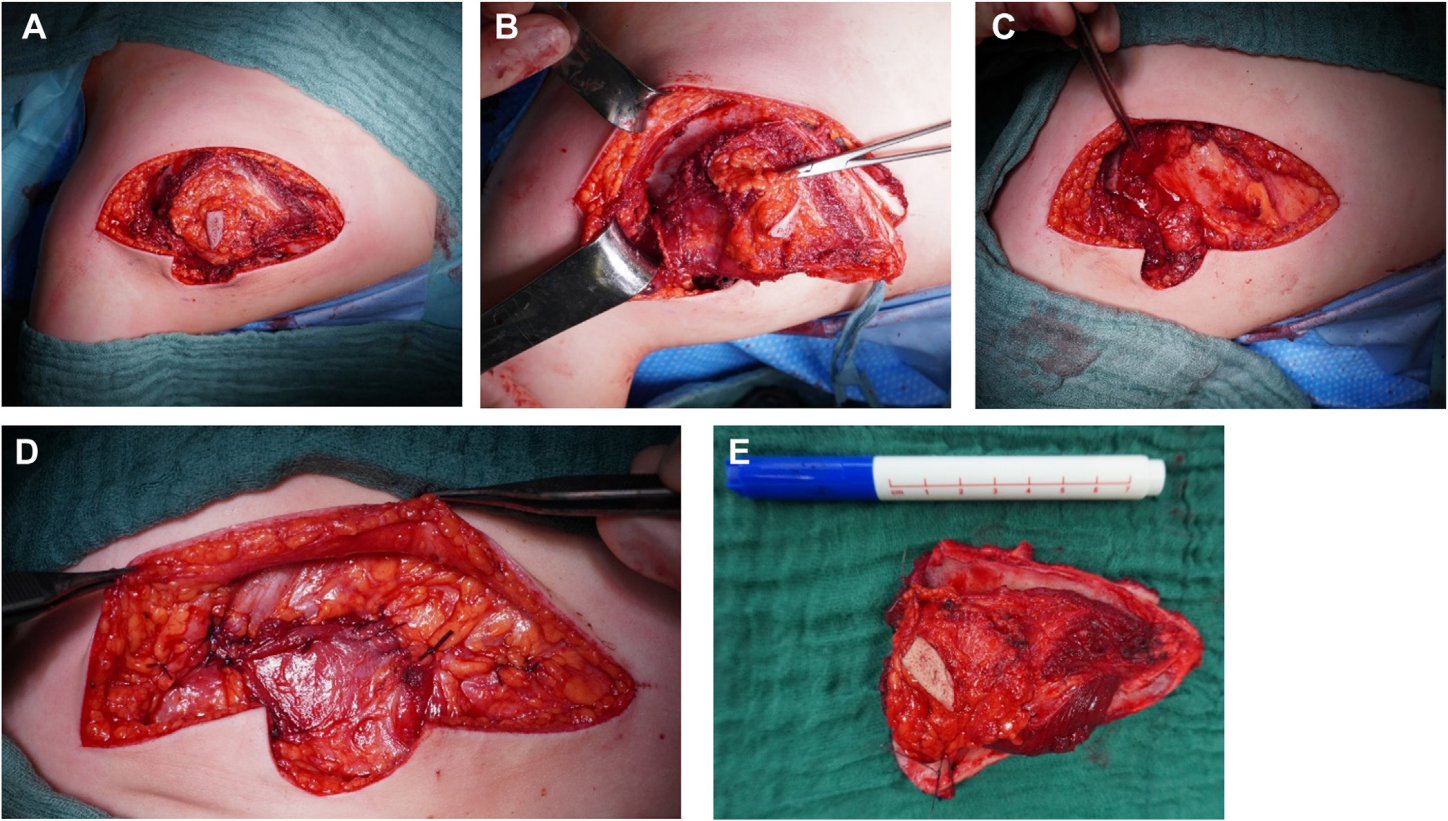
Intraoperative pictures of Malawer II resection performed on patient 1 (prone position, head left side of picture, arm lower side of picture). (**A**) Access to the scapula via Judet incision (including cutaneous muscular biopsy tract). (**B**) Isolation of the scapula and dissection of the surrounding muscle groups. (**C**) Identification of the muscle groups for reconstruction after removal of the scapula body. (**D**) Reconstruction of the muscle layers. (**E**) Resected body of the scapula with adjacent muscles.

All patients underwent chemotherapy according to the EWING 2008 protocol with excellent tumor response and thereafter underwent uneventful (partial) scapulectomy (illustrative intraoperative pictures of patient 1 see Fig. 2). Patients 1 and 2 underwent partial scapulectomy (Malawer II resection/resection of the acromion) and patient 3 underwent total scapulectomy (Malawer III resection). Patients 2 and 3 underwent additional proton beam radiotherapy (total dosage 54 Gy). All patients are alive, last follow-up ranged from 6 months to 10 years. Functional outcome measured by Musculoskeletal Tumor Society (MSTS) was superior in patients 1 and 2 who underwent partial scapulectomy than in patient 3 who underwent total scapulectomy and HS.

## Systematic review of the literature

### Aims and objectives

The aim of the systematic review is to examine the types of resections used in children with scapula tumors and their functional outcome. The objectives were to provide information about the influence of resection type and reconstruction type on the functional outcomes in children.

### Methods

A systematic review and qualitative syntheses were conducted according to the Cochrane guidance for systematic review and meta-analysis and reported in agreement with the preferred reporting items for systematic reviews and meta-analysis guidelines.^23^

#### Search strategy

Based on the findings of a prospective exploratory study by Bramer et al, a systematic search was conducted including the following databases: PubMed, Embase, and Web of Science (38). The search strategy included the keywords scapula, tumor, and scapulectomy. Medical subject headings for each keyword were used. Furthermore, truncations on the keywords were used to expand the search results. A validated pediatric (defined as age <18 years) search filter was used.^15^ After the removal of duplicates, 2 reviewers screened the search results and applied the eligibility criteria listed below to identify relevant articles, with a third reviewer adjudicating as necessary. Articles that were potentially relevant were sought for retrieval and included in full text review. The review process mentioned above was also applied for the full text screening.

#### Eligibility criteria systematic review

Population, Intervention, Comparison, Outcomes, and Studies was used to formulate eligibility criteria:

• P (Population): Children (defined as age <18 years).
• I (Intervention): (Partial) Scapulectomy (Malawer classification II/III) with or without a reconstructive procedure.^16^
• C (Comparison): Influence of resection type and reconstructive procedure on outcomes.
• O (Outcomes): Functional outcome according to the MSTS scoring system.^5^
• S (Studies): Due to the anticipated limited primary data, all types of studies, except systematic reviews, were included.

Studies that included both adults and children but presented data in a pooled manner without specifying functional outcomes of the included children were excluded.

#### Data extraction and analysis

The following data were extracted from the articles: date of publication, methodology, number of cases, age of children, diagnosis, type of scapula resection, reconstruction type, complications, oncologic outcome, functional outcome, and duration of follow-up. The data were managed using Microsoft Excel. A qualitative and quantitative synthesis was performed. Normal distribution of the MSTS scores was confirmed by performing a 2-sample Kolmogorov–Smirnov test. Therefore, a t-test was performed to test for statistical significance in functional outcomes between the different treatment groups.

#### Quality assessment of studies

Like the process of the article selection, 2 independent reviewers assessed the quality of the articles selected, with a third reviewer acting as an umpire. The Joanna Briggs checklist for case reports and case series was used to evaluate the eligible articles. Article quality was classified according to the percentage of criteria they met on the Joanna Briggs checklist (good >80%, fair 50%-79%, and poor <50%).^24^

#### Risk of bias and missing data

The Joanna Briggs checklist for case reports and case series was used to assess for missing data of the eligible studies. If the quality was deemed poor or fair, the study was excluded.^24^ Functional outcome was measured using the validated MSTS scoring system. Since this scoring system not only incorporated objective but also subjective parameters to assess functional outcome, there is an inherited risk of assessment bias. However, since the MSTS is a validating scoring system, the risk of bias was deemed low.^5^

## Results

### Study selection

**Figure 3:**
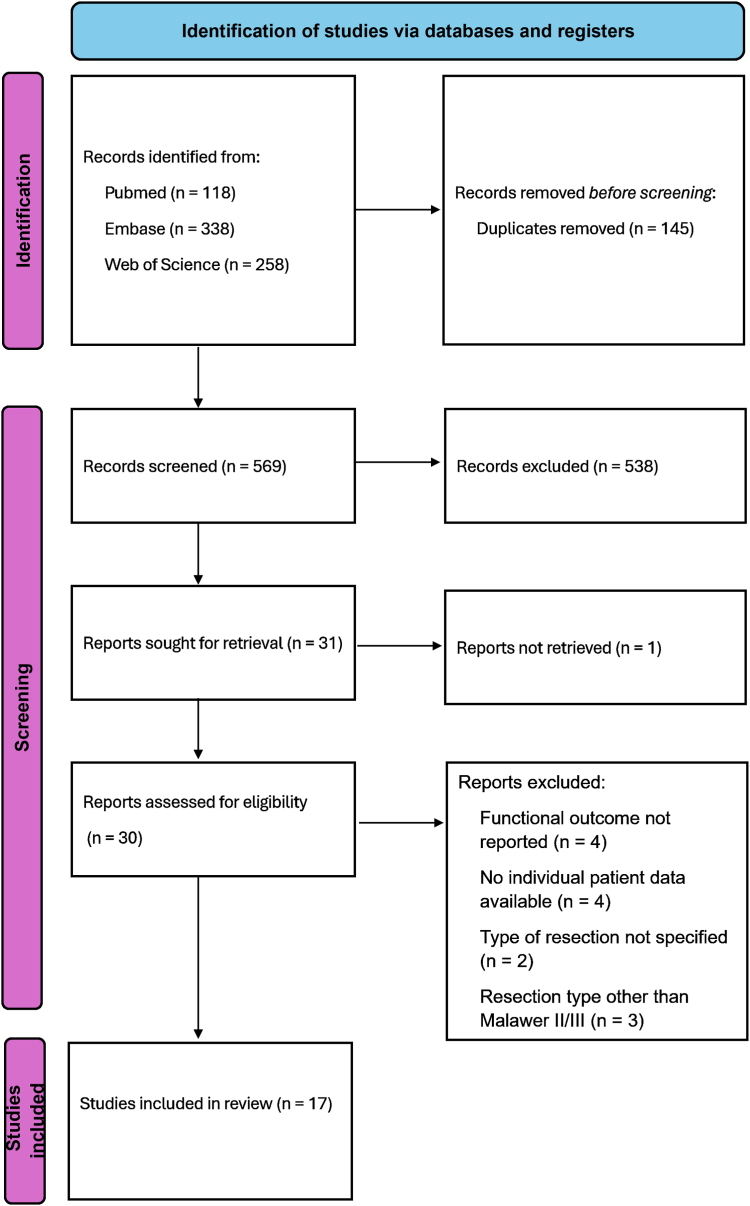
PRISMA flowchart of the screening process of studies included in this systematic review. *PRISMA*, preferred reporting items for systematic reviews and meta-analyses.

The search generated 714 results. After the above-mentioned screening process, 17 studies were deemed eligible to be included in the analysis (Fig. 3 and Table II).

**Table II:** Included studies.

Author/publication, y	Study type	Patients
Beltrami et al, 2018^1^	Case series	1
El Ghoneimy et al, 2018^4^	Case series	9
Hargiss et al, 2022^7^	Case series	3
Hoornenborg et al, 2013^10^	Case report	1
Jamshidi et al, 2020^12^	Case series	2
Kang et al, 2023^14^	Case series	1
Malik et al, 2020^17^	Case series	7
Mayil Vahanan et al, 2007^19^	Case series	8
Mimata et al, 2018^20^	Case series	1
Min et al, 2017^21^	Case series	3
Schmalzl et al, 2018^29^	Case report	1
Schoch et al, 2016^30^	Case series	1
Theologis et al, 2006^32^	Case report	1
Uchida et al, 2009^33^	Case report	1
Wong et al, 2023^37^	Case series	1
Zhang et al, 2009^38^	Case series	1
Zubairi et al, 2021^39^	Case series	5

### Methodology of included studies and data quality

The included studies were either case reports or case series. Applying the Joanna Briggs checklist for case studies and series, all studies were deemed good quality. All included studies used the MSTS score to evaluate the postoperative functional outcome; hence, this test was also chosen to assess the functional outcome in this systematic review.

### Qualitative data analysis

**Figure 4:**
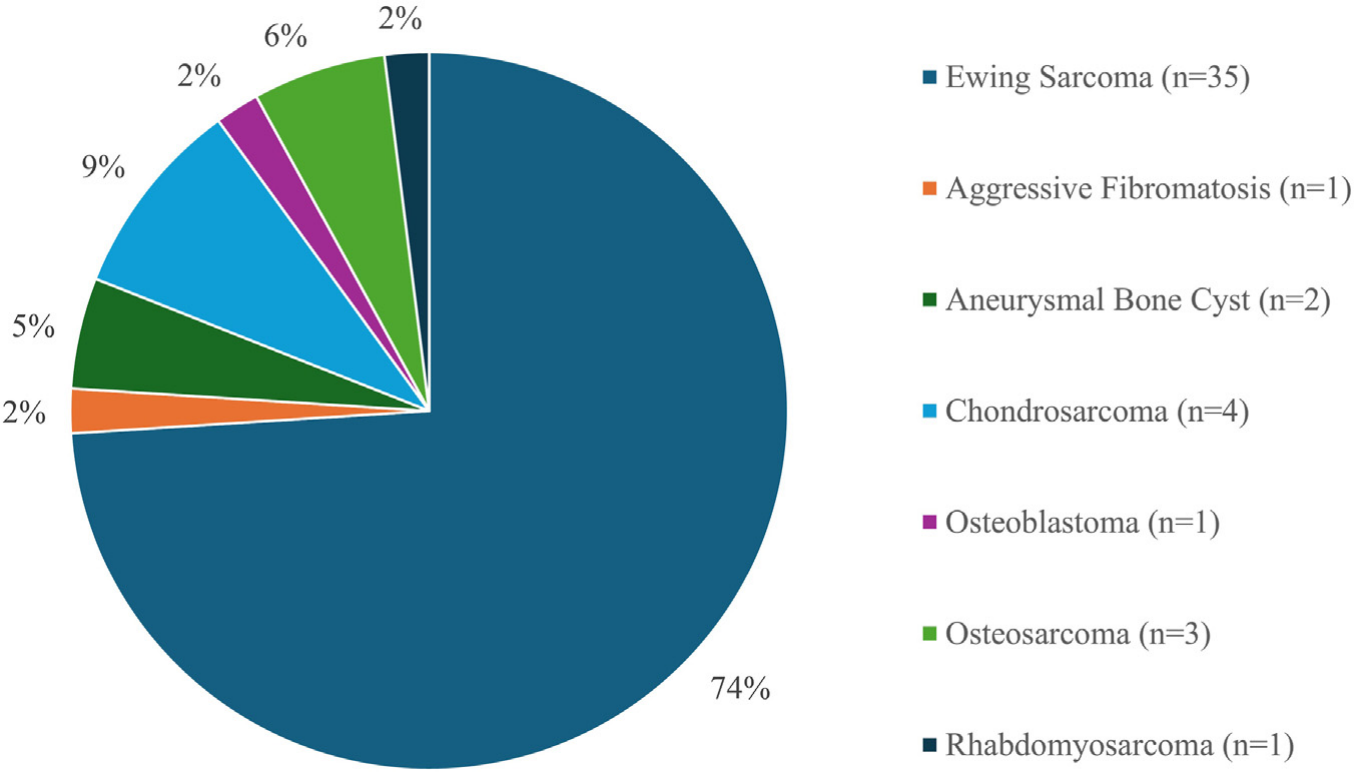
Types of scapular tumor of the 47 patients included.

The 17 case reports and case series included 47 patients (Table II).^12^^,^^14^^,^^17^^,^^19^, ^20^, ^21^^,^^29^^,^^30^^,^^32^^,^^33^^,^^39^ The mean age was 11 years (range 1-17 years) with 65% (n = 31) of the patient population being male (Table III). Figure 2 describes the malignancies arising from the scapula. By far, the most common malignancy was ES (n = 35), which accounted for 74% of all tumors described in this patient collective (Fig. 4). Of the 47 patients, 14 (30%) were treated by glenoid-preserving Malawer II scapular resection and the other 70% were treated by total scapulectomy (Malawer III resection). In total, 16 reconstructive procedures other than HS were performed; most of them (75%) in patients who underwent total scapulectomy. Reconstruction was done either by endoprosthesis (3D-printed/conventional endoprosthesis/allograft) (n = 6) or ECI+RP of the native scapula (n = 10). Of the patients undergoing ECI+RP as reconstructive procedure, 50% of reimplants showed signs of partial reabsorption (scapular body) at the last follow-up. Mean follow-up was 57 months (range 3-215 months), during which 4 patients (2 in the ECI+RP group and 2 in the nonreconstructed group) developed grade I complications (Clavien–Dindo classification).^3^ Six patients died due to the underlying malignancy. MSTS scores and percentage for the individual resection and treatment groups are given in Table III.

**Table III:** Patient characteristics.

No. of patients	m/f	Age [y] (range)	Follow-up [mo] (range)	Alive/dead	Resection (Malawer classification)	Reconstruction	Type of reconstruction	MSTS score (range)	MSTS % (range)
47	31/16	mean = 11 (1-17)	mean = 57 (3-215)	41/6	II = 14	yes = 4	EP = 3	Mean = 25 (22-28)	Mean = 81 (73-93)
							ECI + RP = 1		
						no = 10	-	Mean = 24 (20-30)	Mean = 81 (67-100)
					III = 33	yes (other than HS) = 12	EP = 3	Mean = 26 (21-30)	Mean = 86 (67-100)
							ECI + RP = 9		
						yes (HS) = 21	HS = 21	Mean = 23 (13-27)	Mean = 71 (43-90)

*MSTS*, Musculoskeletal Tumor Society; *EP*, endoprosthesis; *ECI + RP*, extracorporeal irradiation and reimplantation; *HS*, humeral suspension.

### Quantitative data analysis

A 2-sample Kolmogorov–Smirnov test was performed and confirmed normal distribution of the MSTS scores (maximum deviation: 0.184, critical value for a sample of 47: 0.198). Therefore, a t-test (critical *P* value = .05) was deemed appropriate for further statistical testing. The MSTS scores between patients undergoing Malawer II and Malawer III resection did not show statistically significant difference (*P* value .25). Overall, children who underwent Malawer II or Malawer III resection had a significant better functional outcome if they underwent a reconstructive procedure other than HS compared to HS reconstruction (*P* value = .003) Further subanalysis showed that patients who underwent Malawer III resection and a reconstruction other than HS had a significant better functional outcome than those who did undergo HS after Malawer III resection (*P* value = .0007). No significant difference between the MSTS scores of patients who underwent Malawer II resection with or without reconstruction (*P* value = .89) was seen. Furthermore, no significant difference between patients who underwent Malawer II resection without reconstruction and patients who underwent Malawer III resection with reconstruction other than HS (*P* value = .25) was seen. However, patients undergoing Malawer II resection without a reconstructive procedure had a significant better functional outcome than patients undergoing Malawer III resection and HS reconstruction (*P* value = .024). Furthermore, no significant difference was found between patients who underwent either Malawer II or III resection with reconstruction other than HS (*P* value = .47). No statistically significant difference in functional outcome was seen between reconstructive procedures other than HS (endoprosthesis/allograft and ECI+RP) (*P* value = .28). Regarding the group of patients who underwent reconstruction by ECI+RP, no significant difference in MSTS scores were found between patients who had partial scapular reabsorption or those who did not. (*P* value = .58) (Table IV).

**Table IV:** Synthesis of the quantitative analysis.

Compared groups	*P* value	Conclusion
Malawer II/Malawer III	.25	Overall, no significant functional difference in outcome between resection types (possible explanation see discussion).
Malawer II or III RoTHS/Malawer II or III WoR or HS	.003	Overall, a reconstructive procedure other than HS leads to a functional better outcome in Malawer II or III resection.
Malawer III RotHS/Malawer III HS	.0007	Reconstructions other than HS led to a better functional outcome than HS after Malawer III resection.
Malawer II RoTHS/Malawer II WoR	.89	In Malawer II resection, reconstructive procedures do not lead to a favorable functional outcome.
Malaer II WoR/Malawer III RotHS	.25	If after Malawer III resection, reconstructions other than HS are done functional outcome is equal to Malawer II resections.
Malaer II WoR/Malawer III HS	.024	HS after Malawer III resection has a significant worse functional outcome than Malawer II resection.
Endoprosthesis/ECI + RP	.28	No difference in functional outcome has been found between different reconstructive procedures other than HS.
ECI + RP with partial reabsorption/ECI + RP without partial reabsorption	.58	Partial reabsorption does not lead to an inferior functional outcome.

*RotHS*, reconstruction other than humeral suspension; *WoR*, without reconstruction; *HS*, humeral suspension; *ECI + RP*, extracorporeal irradiation and reimplantation.

## Discussion

The single-center experience and systematic review aimed to provide information on functional outcomes of pediatric patients with scapular tumors undergoing Malawer II or III resection for scapular tumors and different reconstructive procedures.

The 17 papers deemed eligible for inclusion provided information about 47 pediatric (age 1-17 years) patients. In this patient cohort, the most common tumor was by far ES (74%) followed by chondrosarcoma (9%). This contrast with the described etiological pattern of pediatric scapula tumors, where benign tumors like osteochondroma are most frequently seen.^26^^,^^13^

However, other reports focusing on scapulectomy in children found the same distribution of etiology, with chondrosarcomas being most common in adolescents and ESs in smaller children.^19^ Therefore, the different etiological pattern may be due to the design of this study to include only patients undergoing (partial) scapulectomy.

In recent years, limb salvage surgery has become standard practice for most patients with tumors of the scapula. However, depending on the location of the primary tumor a specific shoulder girdle resection (classified by Malawer) is indicated. Although Malawer II resection preserves the glenoid, in Malawer III resection, a total scapulectomy is performed which results in considerable deterioration of the shoulder function.^1^^,^^16^^,^^28^

A total of 14 Malawer II and 33 Malawer III resections were included in this cohort. A reconstructive procedure was performed only on 4 of 14 (29%) patients who underwent Malawer II resection, whereas, 12 out of 33 (36%) patients who underwent Malawer III resection underwent a reconstructive procedure (nonHS). In adults, use of reconstructive surgery other than HS (endoprosthesis/allograft/etc.) has been proven to improve functional outcome.^2^^,^^8^^,^^9^^,^^27^ The higher rate of reconstruction other than HS in the Malawer III group could explain the finding that there was no overall significant difference in MSTS scores between Malawer II and III patients. This is backed up by the further analysis that found the following: Firstly, patients who underwent Malawer III resection and a reconstruction other than HS had a significant better functional outcome than those who did undergo HS after Malawer III resection; secondly, no significant difference in MSTS scores was found between patients who underwent a reconstruction other than HS after either Malawer II or III resection. Furthermore, patients undergoing Malawer II resection without a reconstructive procedure had a significant better functional outcome than patients undergoing Malawer III resection and HS reconstruction.

These findings stress that, in patients undergoing Malawer III resection, a reconstructive surgery other than HS facilitates preservation of shoulder function, which is in consistency with findings in adults undergoing scapular resection and reconstruction.^2^^,^^8^^,^^27^^,^^35^

There was no significant functional difference between Malawer II patients who underwent a reconstruction and the ones who did not. Therefore, one can assume that the glenoid plays the most vital part in preserving shoulder function and resection of the scapular body in children can be compensated by muscle reconstruction, which is in line with the existing literature concerning adults.^4^^,^^19^^,^^28^

In the last decades, significant advances have been made in reconstruction of the scapula in adults and reconstructive techniques other than HS have become the procedure of choice if remaining soft tissue permits it.^6^^,^^35^^,^^36^ However, of the available reconstructive techniques (allograft, ECI+RP, and endoprosthesis, either 3D or conventional), none has emerged as the optimal procedure so far.^2^^,^^9^^,^^22^^,^^37^^,^^38^ This study as well did not find any significant functional difference between the different reconstructive procedures other than HS.

Regarding ECI+RP, an established reconstruction method but seldom reported for the scapula, a common drawback is (partial) reabsorption. The latter was found in 60% of patients undergoing this reconstructive procedure included in this study as well.^2^^,^^9^ However, no statistically significant difference in functional outcome was seen between grafts had been partially reabsorbed and the ones that had not. Therefore, it seems that as long as the glenoid remains intact, no functional deficit results from partial reabsorption. Our patient series is in line with the conclusions drawn from the systematic review of the literature.

## Limitations

As scapular tumors are rare in children and adolescents, this systematic review, although including studies written in languages other than English, could not include more than 47 patients. Included studies were retrospective in nature and limited to case reports and case series with varying follow-up periods. Especially in the pediatric population where oncological survival might be up to several decades, longer follow-up periods would be desirable to draw a more profound conclusion about the best reconstruction method concerning the scapula. Moreover, the choice of reconstructive procedure used may be influenced by surgeon’s preference and/or the location of the hospital due to the availability of reconstructive methods (eg, 3D-printed endoprosthesis). Another limitation is that, despite the importance of certain muscle groups to shoulder functionality, no detailed information of the preservation of muscle groups was given in the included studies. Moreover, the exact location of the scapular tumor was not given in the majority of papers; therefore, no objective justification for the performed procedure was possible.

## Conclusion

Scapular tumors in children and adolescents are rare. Reconstructive options are similar to the ones used in adults and show a significantly better functional outcome measured by the MSTS than HS reconstruction in patients who undergo Malawer III resection. As in adults, reconstructive procedures other than HS (endoprosthesis/allograft/extracorporeal irradiation and reconstruction) seem equally effective. If partial absorption occurs in the latter, functional outcome is not hampered as long as the glenoid stays intact. Reconstructive procedures after glenoid-preserving Malawer II resection do not offer any functional benefit.

## Disclaimers:

Funding: No funding was disclosed by the authors.

Conflicts of interest: The authors, their immediate families, and any research foundations with which they are affiliated have not received any financial payments or other benefits from any commercial entity related to the subject of this article.
